# Neoadjuvant radiotherapy for locoregional Siewert type II gastroesophageal junction adenocarcinoma: A propensity scores matching analysis

**DOI:** 10.1371/journal.pone.0251555

**Published:** 2021-05-12

**Authors:** Yuan Zhou, MengXiang Tian, Cenap Güngör, Dan Wang

**Affiliations:** 1 Department of General Surgery, Xiangya Hospital, Central South University, Changsha, China; 2 Department of General Visceral and Thoracic Surgery, University Medical Center Hamburg-Eppendorf, Hamburg, Germany; The University of Texas MD Anderson Cancer Center, UNITED STATES

## Abstract

**Objective:**

To analyze the effect of neoadjuvant radiotherapy (nRT) on prognosis in patients with locoregional Siewert type II gastroesophageal junction adenocarcinoma (GEA).

**Method:**

All patients pathologically diagnosed as Siewert type II GEA between 2004 and 2015 were retrieved from the Surveillance, Epidemiology and Final Results (SEER) database. We analyzed the impact of different treatment regimens on the prognosis in each stage. Survival analysis was performed by Kaplan-Meier (K-M) method. Multivariate Cox model and propensity score matching was further used to verify the results.

**Results:**

4,160 patients were included in this study. The efficacy of nRT was superior to that of adjuvant radiotherapy (aRT) (p = 0.048), which was the same as that of surgery combined with chemotherapy (p = 0.836), but inferior to the overall survival (OS) of surgical treatment alone (p<0.001) in T1-2N0M0 patients. Patients receiving nRT had distinctly better survival than those receiving surgical treatment alone (p = 0.008), but had similar survival compared with patients treated with aRT (p = 0.989) or surgery combined with chemotherapy (p = 0.205) in the T3N0/T1-3N+M0 subgroup. The efficacy of nRT is clearly stronger than that of surgical therapy alone (p<0.001), surgery combined with chemotherapy (p<0.001), and aRT (p = 0.008) in patients with T4 stage. The survival analysis results were consistent before and after propensity score matching.

**Conclusion:**

In these carefully selected patients, the present study made the following recommendations: nRT can improve the prognosis of patients with T3N0M0/T1-3N+M0 and T4 Siewert type II GEA, and it seems to be a better treatment for T4 patients. Surgery alone seems to be sufficient, and nRT is not conducive to prolonging the survival of Siewert II GEA patients with T1-2N0M0 stage. Of course, further prospective trials are needed to verify this conclusion.

## Introduction

It was estimated that about 18,000 new cases and 13,000 deaths from esophageal cancer occur in the United States in 2020 [1]. Adenocarcinoma, accounting for 75% of esophagus cancers, is mainly located in the lower esophagus and gastroesophageal junction (GEJ) in the US, and its incidence has raised significantly since the 1970s [2]. Most patients with gastroesophageal junction adenocarcinoma (GEA) often have a terrible prognosis, with a 5-year survival of less than 25%, because of late-stage diagnosis and rapid spread [3]. Siewert classification is ground on the anatomical distance between the tumor center and the GEJ, which divides GEA into three grouplets: Siewert type I, type II, and type III [4] and is now widely used in clinical practice. Siewert type I (distal esophageal adenocarcinoma) originates from the specialized intestinal area of the esophagus (such as Barrett’s esophagus), which can infiltrate the esophagus-gastric junction from above(located 1–5 cm above the GEJ); Siewert type II (cardia cancer) originates from the junction of the esophagus and stomach(located 1cm above the GEJ to 2cm below); Siewert type III (subcardial gastric carcinoma) refers to the esophagogastric junction and the distal esophagus are infiltrated from the bottom inward(located 2–5 cm below the GEJ) [4]. It has been agreed, clinically, that type I and III GEA can be staged and treated with reference to carcinoma of esophagus and gastric cancer, respectively, due to the similarity in pathology and biological behavior [5]. Although the latest TNM staging system (8th edition) classifies Siewert type II as esophageal cancer, it is difficult to determine whether the origin is gastric cancer or esophageal cancer, so the optimal treatment has been controversial.

At present, surgery is the basis for the treatment of Siewert type II GEA patients without distant metastasis, and the pivotal goal is to achieve radical resection. However, the treatment outcome of only surgery is often disappointing, which has prompted the development of multimodal therapy for GEA [6]. Neoadjuvant chemotherapy is superior to surgical treatment alone for resectable esophagus cancer and GEA in some randomized clinical trials [7,8] and has been widely used clinically. Currently, neoadjuvant chemotherapy for type II GEA is mainly aimed at patients with locally advanced tumors that invade the gastric wall to a depth of T3 or T4, and it is expected that surgical resection is difficult or cannot achieve R0 resection. Its main chemotherapy regimen mainly refers to the neoadjuvant chemotherapy regimen for gastric cancer [9]. In addition, neoadjuvant radiotherapy (nRT) is mainly used to control local disease and improve marginal negative resection. However, because of the contradictory results of some clinical trials [10–12], whether patients with GEA can benefit from nRT is still inconclusive and needs further study.

Moreover, the necessity of nRT for the treatment of cavity organ tumors is controversial and some studies have shown that nRT does not improve the survival of these patients [13]. In addition, radiotherapy may lead to edema, fibrosis, and normal tissue structure disorder in the surrounding tissues of the tumor, which makes it difficult for the surgeon to perform radical resection and increases the probability of postoperative complications [14,15]. Therefore, some researchers have proposed to exclude radiotherapy in the treatment of rectal cancer [14]. Does the idea of abandoning radiotherapy apply to all cavity organ tumors? Therefore, this study aims to explore the significance of nRT for Siewert II tumor patients, so as to propose individualized treatment strategies.

This study tried to use the information from the specific cancer database, the Surveillance, Epidemiology, and End Results (SEER) database, and divided the treatment strategies into surgery-only cohort, nRT cohort, adjuvant radiotherapy (aRT) cohort, and surgery plus chemotherapy cohort to analyze the influence of nRT on the prognosis of non-metastatic Siewert II GEA patients.

## Methods

### Data provenience

The present study extracted GEA cases from the database through SEER Stat software. The SEER database incorporates basic demographic data and some clinical characteristics, mainly from 18 cancer registration centers, accounting for about 28% of the American populace [16]. This study is based on a retrospective analysis in the SEER database and has no identifiable patient information in the database, which is anonymous. Therefore, written informed consent is not required in this study. The study is based on the ethical standards of the Helsinki Declaration as well as national and international norms.

### Patient population

**Fig 1:**
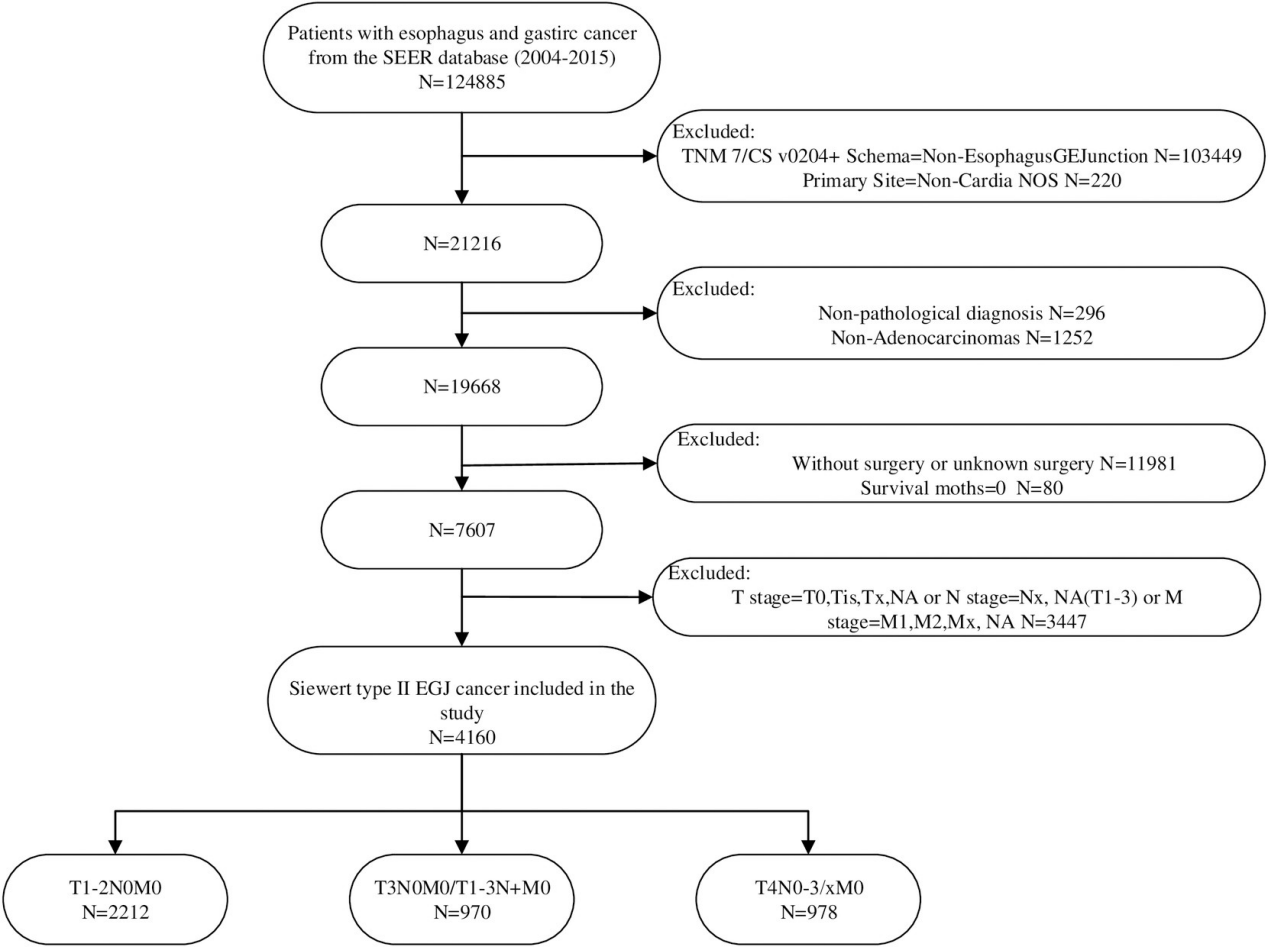
Inclusion and exclusion procedures for Siewert type II EGA patients from SEER database.

GEA patients are derived from the up-to-date version of the SEER database with additional treatment fields (SEER 18, 1973–2014 varying), which was based on the November 2016 submission and was released in March 2018. Although there is no specific Siewert classification in this database, we classified cancers whose ‘Primary Site’ is ‘C16.0-Cardia NOS’ and ‘CS v0204+ Schema’ is ‘EsophagusGEJunction’ as Siewert type II GEA referring to previous studies [17,18]. We retrieved all Siewert type II GEA patients diagnosed pathologically between the years 2004–2015 from the SEER database. The extracted information mainly incorporated basic information (age, sex, race, insurance, and marital status), specific pathological data (tumor grade, pathological type, TNM stage), treatment information (operation, chemotherapy and radiotherapy), other clinical data (lymph node dissection, tumor size) and follow-up data. The database used the 7th (2010–2105) and 6th (2004–2015) TNM staging systems from 2004 to 2015, so we converted the 6th edition to the 7th edition based on CS Extension and CS Lymph Nodes. We selected patients with surgery code 30–80 from the SEER database, which means that these patients have received at least partial gastrectomy. This study only included patients with non-metastatic GEA (T1-4NxM0), and the specific process of inclusion and exclusion can be seen in **Fig 1**. Both neoadjuvant radiotherapy and adjuvant radiotherapy patients received chemotherapy after screening. We divided the treatment strategy into four cohorts: surgery cohort (patients only received surgery), surgery combined with chemotherapy cohort (patients underwent surgery and chemotherapy, without radiotherapy), and nRT cohort (patients treated with nRT and surgical treatment, with chemotherapy), aRT cohort (patients received surgical treatment combined with chemotherapy and aRT).

### Statistical analyses

Overall survival (OS), that is, from the time of diagnosis of GEA to death or the last follow-up, was the principal end point of the study. First, the chi-square test was applied to compare patient characteristics between treatment groups. The log-rank test was utilized to estimate and analyze patients’ 3-year, 5-year, and median OS. We performed univariate and multivariate Cox models to analyze patients in each group and to determine risk ratios (HR) and 95% confidence intervals (CI). This study uses propensity score matching to reduce the possibility of various treatment selection biases. Insurance, marital status, age, race, gender, pathological type, tumor grade, T stage, RNE and tumor size were used as matching criteria to estimate propensity scores in the T1-2N0M0 group. In addition to the above indicators, N stage was added as a matching criterion to estimate the propensity score in the T3N0M0/T1-3N+M0 and T4N0-3/xM0 group. Propensity score matching pairs were identified without replacement using a 1:1 nearest neighbor matching algorithm with caliper width determined by the recommendation from Austin (0.002 of the standard deviation of the logit of the PSs). All statistical analyses in this study were run under SPSS 26.0 software, and the inspection level of all statistical analyses was set to p-value less than 0.05. In addition, GraphPad Prism 8 software was used to draw the Kaplan-Meier (K-M) survival curve.

## Result

### Basic characteristics of the patients

Overall, after screening, 4,160 GEA patients were finally incorporated in this study. nRT was carried out in 24.57% (1,022) of the total population and around 12.19% (507) underwent aRT. About 45.70% (1,901) of patients received chemotherapy. The majority of the study group were married white, and 56.80% were older than 65. Most of the patients studied were in T1 stage, accounting for 42.54% (1,770), and 23.51% (978) were in T4 stage. Patients with tumor grades III and IV account for a high proportion (41.26%) of the population. The basic clinical and pathological features of the subjects were displayed in **Table 1**.

**Table 1:** The basic clinicopathological features of patients with Siewert type II EGA.

Features	T1-2N0M0(N = 2212)	T3N0M0/T1-3N+M0(N = 970)	T4N0-3/xM0 (N = 978)
	Number (%)	Number (%)	Number (%)
Insurance Recode			
No/Unknown	660(29.84%)	221(22.78%)	498(50.92%)
Insured	1552(70.16%)	749(77.22%)	480(49.08%)
Marital status			
Single/Unknown	730(33.00%)	317(32.68%)	340(34.76%%)
Married	1482(67.00%)	653(67.32%)	638(65.24%)
Race			
Non-whites	212(9.58%)	103(10.62%)	130(13.29%)
White	2000(90.42%)	867(89.38%)	848(86.71%)
Age			
<65	834(37.70%)	429(44.23%)	534(54.60%)
≥65	1378(62.30%)	541(55.77%)	444(45.40%)
Sex			
Female	510(23.06%)	186(19.18%)	218(22.29%)
Male	1702(76.94%)	784(80.82%)	760(77.71%)
Histology			
Adenocarcinomas	2056(92.95%)	809(83.40%)	784(80.16%)
Cystic, mucinous and serous neoplasms	156(7.05%)	161(16.60%)	194(19.84%)
Grade			
I	310(14.01%)	52(5.36%)	34(3.48%)
II	927(41.91%)	325(33.51%)	257(26.28%)
III/IV	636(28.75%)	519(53.51%)	636(65.03%)
Unknown	339(15.33%)	74(7.62%)	51(5.21%)
T stage			
T1	1760(79.57%)	10(1.03%)	-
T2	452(20.43%)	27(2.78%)	-
T3	-	933(96.19%)	-
T4	-	-	978(100%)
N stage			
N0	2212(100%)	746(76.90%)	185(18.92%)
N1	-	104(10.72%)	40(4.09%)
N2	-	70(7.22%)	13(1.33%)
N3	-	50(5.16%)	15(1.53%)
Unknown	-	-	725(74.13%)
Therapy			
Surgery alone	1721(77.80%)	240(24.74%)	235(24.03%)
Surgery + chemotherapy	114(5.15%)	139(14.34%)	182(18.61%)
nRT	269(12.17%)	473(48.76%)	280(28.63%)
aRT	108(4.88%)	118(12.16%)	281(28.73%)
RNE			
<15	1537(69.48%)	513(52.89%)	503(51.43%)
≥15	638(28.85%)	441(45.46%)	456(46.63%)
Unknown	37(1.67%)	16(1.65%)	19(1.93%)
Tumor size			
<3cm	883(39.92%)	82(8.45%)	29(2.97%)
≥3cm and <5cm	669(30.24%)	441(45.46%)	360(36.81%)
≥5cm	120(5.42%)	301(31.04%)	431(44.07%)
Unknown	540(24.42%)	146(15.05%)	158(16.15%)

Abbreviations GEA: Gastroesophageal junction adenocarcinoma; nRT: Neoadjuvant radiotherapy; aRT: Adjuvant radiotherapy; RNE: Regional nodes examined.

### Survival analysis before PSM

**Fig 2:**
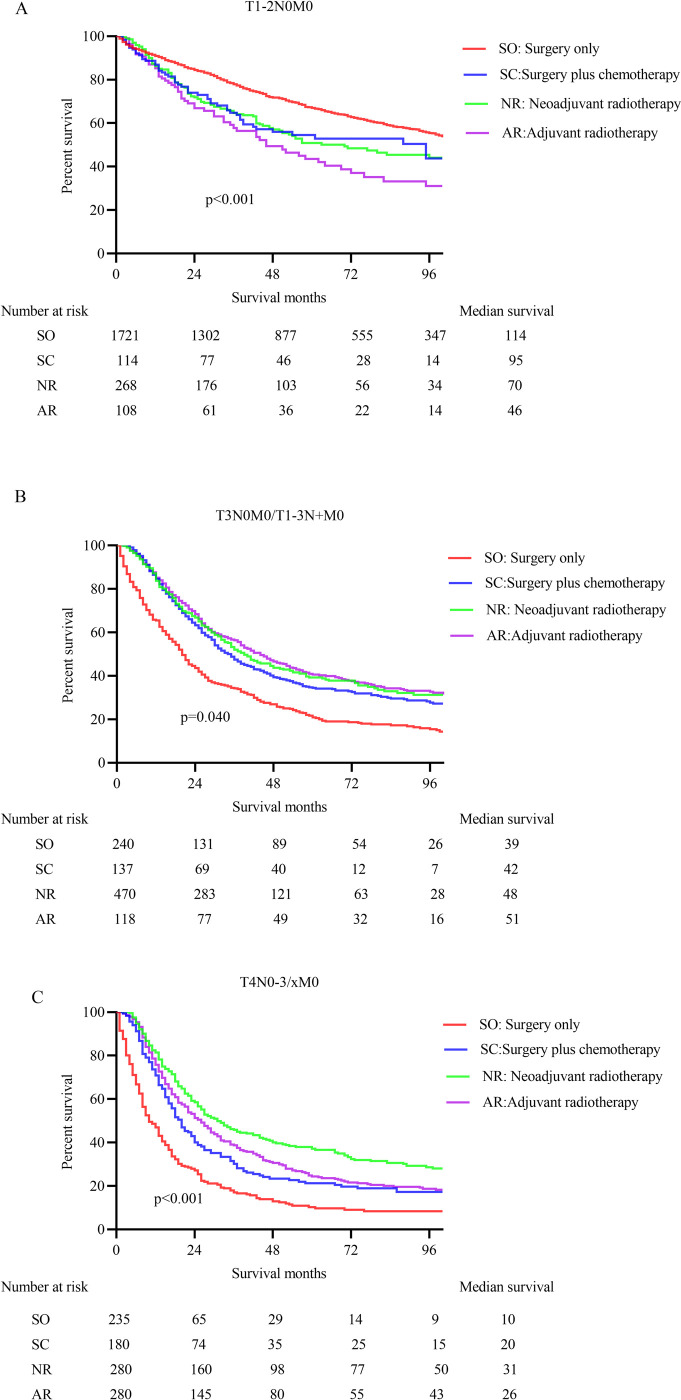
The overall survival estimated with the Kaplan-Meier method for non- metastatic Siewert type II EGA patients. A. The OS analysis of different treatment methods in T1-2N0M0 stage (p<0.001); B. The OS analysis of different treatment methods in T3N0/T1-3N+M0 stage (p = 0.040); C. The OS analysis of different treatment methods in T4 stage (p<0.001).

First, the results of Cox regression model analysis in the entire group showed that age, marital and insurance status, grade, pathological type, T stage, N stage, treatment mode, and regional lymph node examination (RNE) are closely related to OS (**Table 2**). The tumor outcomes of nRT, aRT, and surgery combined with chemotherapy were significantly better than surgery alone for patients with non-stage IV Siewert type II GEA (p = 0.004). The influences of various therapy modes on the prognosis of patients were further analyzed in subgroups of different stages. The K-M curve of OS in each stage was manifested in **Fig 2**.

**Table 2:** The Cox regression model analysis for OS of all Siewert type II GEA patients.

		Univariate analysis	Multivariate analysis		
Features	classification	P	HR	95%CI	P
Insurance status		<0.001			<0.001
	No/unknown		Reference	Reference	Reference
	Insured		0.848	0.775–0.929	<0.001
Marital status		<0.001			<0.001
	Single/Unknown		Reference	Reference	Reference
	Married		0.832	0.761–0.908	<0.001
Age, years		<0.001			<0.001
	<65		Reference	Reference	Reference
	≥65		1.773	1.619–1.941	<0.001
Race recode		0.068			
	No-whites				
	White				
Sex		0.373			
	Female				
	Male				
Histology		<0.001			0.038
	Adenocarcinomas		Reference	Reference	Reference
Cystic, mucinous and serous neoplasms			1.134	1.007–1.278	0.038
Grade		<0.001			<0.001
	I		Reference	Reference	Reference
	II		1.084	0.910–1.290	0.368
	III/IV		1.425	1.197–1.696	<0.001
	Unknown		0.831	0.665–1.038	0.103
T stage		<0.001			<0.001
	T1		Reference	Reference	Reference
	T2		1.590	1.360–1.859	<0.001
	T3		1.816	1.564–2.109	<0.001
	T4		2.260	1.869–2.733	<0.001
N stage		<0.001			<0.001
	N0		Reference	Reference	Reference
	N1		1.550	1.234–1.946	<0.001
	N2		1.914	1.427–2.567	<0.001
	N3		2.892	2.160–3.870	<0.001
	Unknown		1.712	1.439–2.037	<0.001
Treatment methods		<0.001			0.004
	Surgery alone		Reference	Reference	Reference
Surgery plus chemotherapy			0.898	0.757–0.961	0.046
nRT			0.838	0.739–0.949	0.005
aRT			0.824	0.714–0.950	0.008
RNE		0.042			<0.001
	<15		Reference	Reference	Reference
	≥15		0.703	0.641–0.771	<0.001
	Unknown		0.865	0.614–1.217	0.405
Tumor size		<0.001			0.126
	<3cm		Reference	Reference	Reference
	≥3cm and <5cm		1.089	0.950–1.247	0.220
	≥5cm		1.196	1.024–1.397	0.024
	Unknown		1.127	0.973–1.305	0.112

Abbreviations GEA: Gastroesophageal junction adenocarcinoma; nRT: Neoadjuvant radiotherapy; aRT: Adjuvant radiotherapy; RNE: Regional nodes examined; OS: Overall survival; HR: Hazard ratio; CI: Confidence interval.

**Fig 3:**
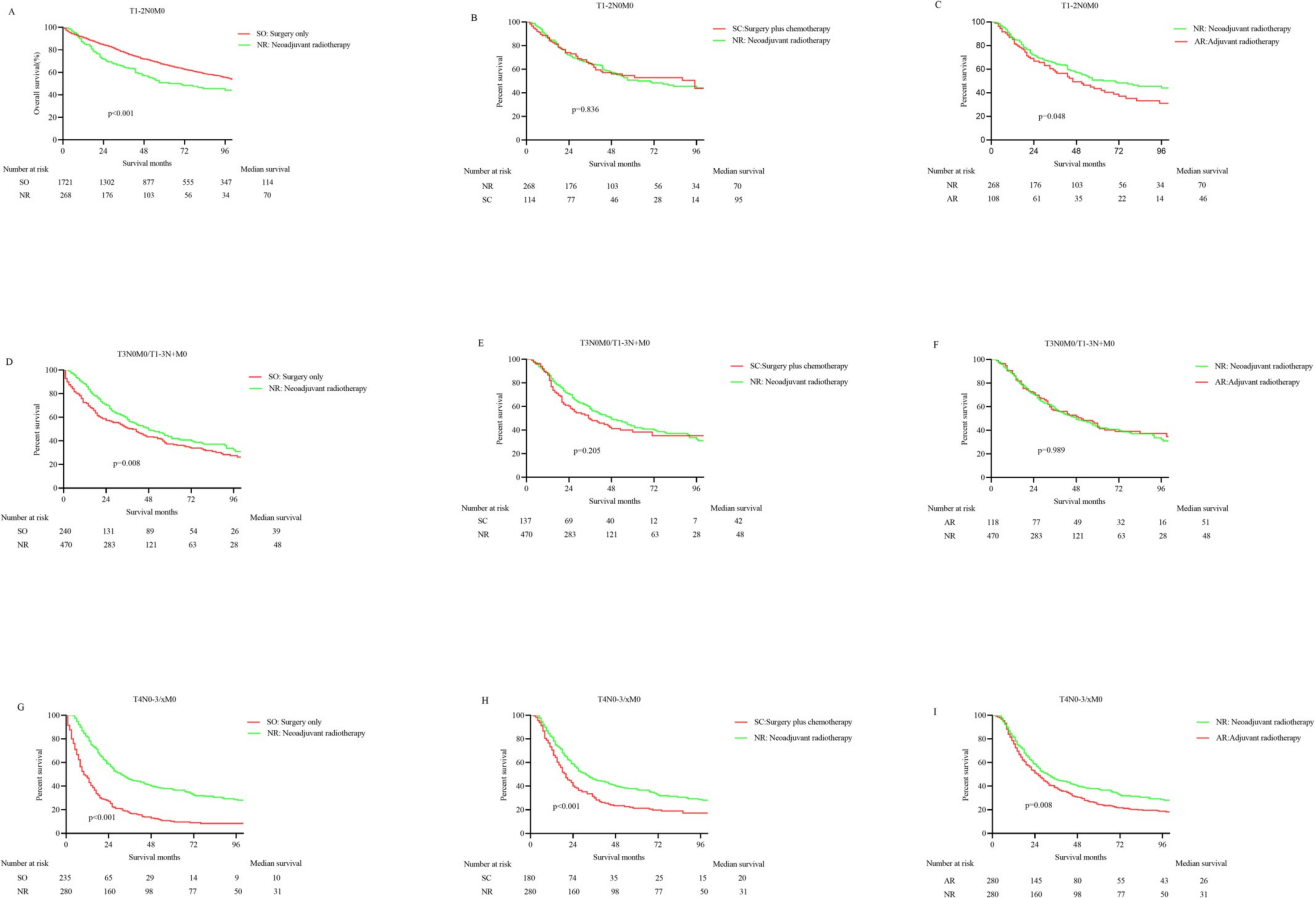
The K-M curves for OS in non- metastatic Siewert type II EGA patients at different stages before PSM. A. Surgery only vs Neoadjuvant radiotherapy in T1-2N0M0; B. Surgery combined with chemotherapy vs Neoadjuvant radiotherapy in T1-2N0M0; C. Neoadjuvant radiotherapy vs Adjuvant radiotherapy in T1-2N0M0; D. Surgery only vs Neoadjuvant radiotherapy in T3N0/T1-3N+M0; E. Surgery combined with chemotherapy vs Neoadjuvant radiotherapy in T3N0/T1-3N+M0; F. Neoadjuvant radiotherapy vs Adjuvant radiotherapy in T3N0/T1-3N+M0; G. Surgery only vs Neoadjuvant radiotherapy in T4; H. Surgery combined with chemotherapy vs Neoadjuvant radiotherapy in T4; I. Neoadjuvant radiotherapy vs Adjuvant radiotherapy in T4.

About 12.16% of patients received nRT in the T1-2N0M0 subgroup. The efficacy of nRT is superior to that of aRT (HR 0.738, 95%CI 0.533–0.920; p = 0.048), and it is the same as that of surgery plus chemotherapy (HR 0.996, 95%CI 0.699–1.336; p = 0.836). Nonetheless, the overall survival of patients who only received surgery was indeed longer than that of nRT in patients with T1-2N0M0 stage (HR 0.674, 95%CI 0.539–0.842; p<0.001) (**Fig 3A–3C**). The median survival was 70, 46, 95, and 114 months for nRT, aRT, surgery combined with chemotherapy, and surgery alone cohorts, respectively (**Table 3**).

**Table 3:** Multivariate Cox analysis of OS with various treatment methods, median survival and 3-year and 5-year OS.

TNM Stage	Treatments	Multivariate HR (95% CI)	P value	Median survival	3-year OS	5-year OS
T1-2N0M0			<0.001			
Only surgery		Reference		114	77.91%	66.94%
Surgery + chemotherapy		1.499(1.121–2.004)	0.006	95	64.82%	54.57%
nRT		1.465(1.195–1.795)	<0.001	70	64.62%	50.81%
aRT		1.829(1.402–2.386)	<0.001	46	57.83%	43.54%
T3N0/T1-3N+M0			0.008			
Only surgery		Reference		39	40.81%	27.39%
Surgery + chemotherapy		0.803(0.503–0.968)	0.041	42	54.66%	40.03%
nRT		0.755(0.612–0.932)	0.009	48	56.94%	42.61%
aRT		0.716(0.534–0.958)	0.025	51	58.06%	43.59%
T4N0-3/xM0			<0.001			
Only surgery		Reference		10	17.06%	9.74%
Surgery + chemotherapy		0.594(0.476–0.740)	<0.001	20	30.16%	21.29%
nRT		0.347(0.263–0.449)	<0.001	31	45.80%	37.08%
aRT		0.584(0.498–0.689)	<0.001	26	38.59%	24.35%

Abbreviations OS: Overall survival; HR: Hazard ratio; CI: Confidence interval; nRT: Neoadjuvant radiotherapy; aRT: Adjuvant radiotherapy.

The nRT was administered to 48.76% of patients in the T3N0/T1-3N+M0 subgroup. The prognosis of patients undergoing nRT distinctly won upon that of patients undergoing surgical treatment alone (HR 0.765, 95%CI 0.621–0.943; p = 0.008), with median survival times of 48 and 39 months, respectively. There was no striking disparity in the survival between nRT and aRT cohort (HR 1.002, 95%CI 0.766–1.311; p = 0.989; Median survival: 51 months) or surgery combined with chemotherapy cohort (HR 1.183, 95%CI 0.898–1.559; p = 0.205; Median survival: 42 months) (**Fig 3D–3F**).

Only 28.63% of patients received nRT in the T4 subgroup, but the efficacy of nRT was markedly superior to that of surgery alone (HR 0.323, 95%CI 0.265–0.392; p<0.001), surgery combined with chemotherapy (HR 0.657, 95%CI 0.523–0.825; p<0.001), and aRT (HR 0.775, 95%CI 0.640–0.938; p = 0.008), with median survival of 31 months, 10 months, 20 months, and 26 months, respectively (**Fig 3G–3I**).

### Survival analysis after PSM

**Fig 4:**
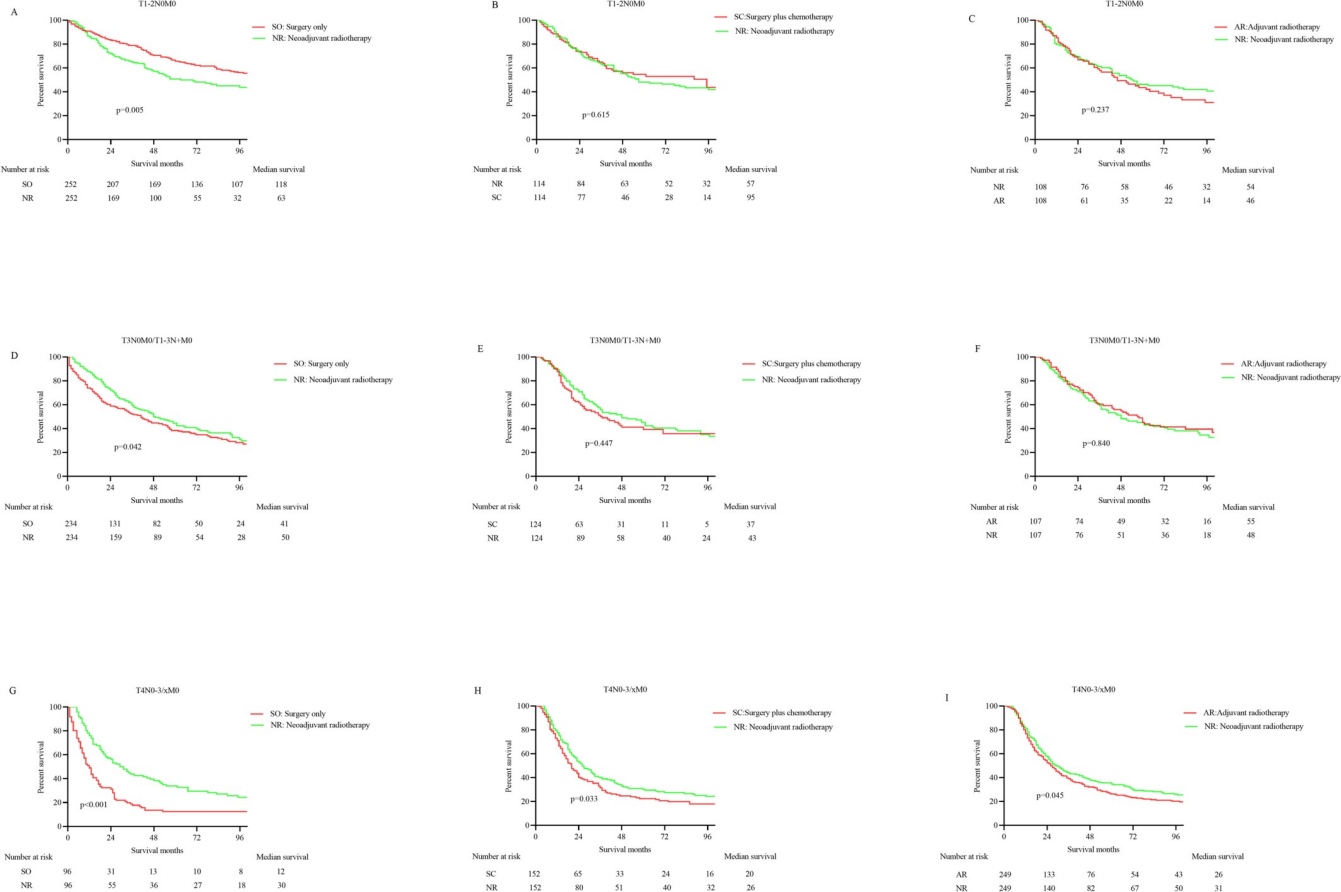
The K-M curves for OS in non- metastatic Siewert type II EGA patients at different stages after PSM. A. Surgery only vs Neoadjuvant radiotherapy in T1-2N0M0; B. Surgery combined with chemotherapy vs Neoadjuvant radiotherapy in T1-2N0M0; C. Neoadjuvant radiotherapy vs Adjuvant radiotherapy in T1-2N0M0; D. Surgery only vs Neoadjuvant radiotherapy in T3N0/T1-3N+M0; E. Surgery combined with chemotherapy vs Neoadjuvant radiotherapy in T3N0/T1-3N+M0; F. Neoadjuvant radiotherapy vs Adjuvant radiotherapy in T3N0/T1-3N+M0; G. Surgery only vs Neoadjuvant radiotherapy in T4; H. Surgery combined with chemotherapy vs Neoadjuvant radiotherapy in T4; I. Neoadjuvant radiotherapy vs Adjuvant radiotherapy in T4.

The multiple 1:1 PSM to compare different treatment regimens created three new comparison subgroups in stage T1-2N0M0 patients: nRT versus surgery alone (n  =  252 pairs), nRT versus surgery combined with chemotherapy (n  =  114 pairs), and nRT versus aRT (n  =  108 pairs). Further K-M analysis found there was no striking disparity between the OS of the nRT cohort and the surgery combined with the chemotherapy cohort (HR 1.096, 95%CI 0.764–1.573; p = 0.615), and the survival advantage compared with the aRT cohort disappeared (HR 1.228, 95%CI 0.866–1.742; p = 0.237), while the survival of the surgical treatment cohort was still significantly superior to the nRT cohort (HR 0.702, 95%CI 0.541–0.909; p = 0.005) (**Fig 4A–4C**).

The PSM analysis of stage T3N0M0/T1-3N+M0 patients, which contained 234 ones per matched group, indicated the OS of nRT was superior to surgery alone (HR 1.256, 95% CI 1.011–1.585; p  =  0.042). In addition, nRT has no obvious survival superiority compared with aRT (HR 0.966, 95% CI 0.685–1.362; p  =  0.840) after matching (n  =  107 pairs). Similarly, no significant disparities in OS could be identified between the nRT and the surgery combined with chemotherapy groups (HR 1.133, 95% CI 0.813–1.580; p  =  0.447) after PSM analysis (n = 124 pairs) (**Fig 4D–4F**).

The 1:1 PSM analysis among stage T4 patients, in which 96 patients treated with nRT were matched to 96 patients undergoing surgery alone, yielded OS favored the nRT cohort (HR 0.523, 95% CI 0.378–0.721; p <0.001). Versus the surgery plus chemotherapy group, the nRT group manifested a distinctly longer survival (HR 0.769, 95% CI 0.596–0.993; p  = 0.033) after PSM analysis (n = 152 pairs). And matched patients with nRT, after matching (n = 249 pairs), were related to a significantly better OS than the aRT cohort (HR 0.752, 95% CI 0.597 to 0.942; p  = 0.045) (**Fig 4G–4I**). The characteristics of patients before and after PSM in each group are shown in S1–S9 Tables.

## Discussion

Surgical resection, as the main treatment for most operable GEA patients, has always been associated with poor survival, which may be due to the relative difficulty of some patients to achieve radical resection or some patients still have distant metastases after radical resection [19]. For this reason, the neoadjuvant therapy has become to be the most shining star in the field of clinical treatment of GEA, including preoperative chemotherapy and radiotherapy. Existing studies have confirmed that neoadjuvant chemotherapy is superior to surgery alone and is a comprehensive treatment method that is easily accepted by GEA patients [20,21]. Although some randomized trials have been conducted so far, the treatment of nRT in GEA patients remains controversial. The CROSS trial is a multiagency phase III clinical trial in which 366 ones with esophageal cancer or GEA were randomly allotted to the surgical-only, preoperative or postoperative chemoradiotherapy groups, confirming that preoperative chemoradiotherapy is superior to surgical treatment alone and that preoperative treatment is the standard treatment [22]. However, the results of a comparative study showed that preoperative radiotherapy did not significantly improve the OS of the lower esophagus and GEA [23]. What’s more, another large retrospective analysis showed that nRT seems to enhance the venture of death among patients with resectable GEA [24].

Hence, we believe that non-metastatic GEA patients should be further staged to discuss the effect of nRT, rather than considered as a whole. First of all, although radical surgical resection and adjuvant treatment provide the possibility of curing localized diseases, most patients with clinical T3 and T4 tumors have a poor prognosis, especially T4 stage [25]. What’s more, if patients with esophageal cancer have lymph node metastasis, the prognosis is generally frustrating, and adjuvant therapy is recommended [26]. In addition, whether induction therapy can improve the survival of patients with early localized disease (T1-2N0M0) is now a fierce controversy [27,28]. With these questions in mind, GEA patients were separated into three subgroups: T1-2N0M0, T3N0/T1-3N+M0, and T4NxM0 to analyze the influence of various therapy options including nRT on the prognosis.

For all we know, our study is the only study to evaluate the impact of nRT on the prognosis of non-metastatic GEA in different stages. Firstly, the results revealed that nRT was detrimental to prolonged survival in T1-2N0M0 patients. Not only for GEA but also for pancreatic cancer, the question whether neoadjuvant therapy should be used in early-stage patients has always been a question. The reason for opposing neoadjuvant therapy for patients with early resectable cancer is that neoadjuvant therapy may cause patients to miss the best opportunity for surgery, making lesions that could be resectable at R0 progress to incurable resection, or even distant metastases [29,30]. Our consequences are confirmed by other retrospective researches, indicating that routine use of neoadjuvant induction therapy may be adverse rather than beneficial to survival in all T1-2N0M0 patients [31]. The real challenge for stage T1-2 esophageal or GEA remains to perfect the precision of the inspection of microscopic lymph node metastases, but currently imaging and endoscopy methods seem to be inadequate [31]. In addition, the T1-2N0M0 stage esophageal cancer or GEA is a localized disease in which the tumor infiltrates into the submucosal layer and may increase the risk of lymph node metastasis, but removal of tumor lesions and local lymph node dissection may be adequate to bring the disease under control, and additional neoadjuvant or adjuvant therapy may have no prognostic benefit [32]. Therefore, combined with our results, only surgery is advised as the main therapy for patients with stage T1-2N0M0 GEA.

The results of a European multicenter retrospective study, which collected data from 30 European centers of patients undergoing esophageal/ GEA surgery, suggest a remarkable survival advantage from nRT for T3N0M0 carcinoma of esophagus [33]. This also further confirms our findings that T3N0M0 should be considered as a locally advanced esophageal cancer like T1-3N+M0, which can benefit from neoadjuvant therapy, but the risk of postoperative complications will not increase significantly. The nRT, aRT, and surgery combined with chemotherapy all can prominently improve OS compared to only surgery for the large subgroup of T3N0/T1-3N+M0 patients, but the best treatment plan still needs further study. In addition, a review also has yielded similar results, showing that multimodal treatment combined with surgery, radiotherapy, and neoadjuvant therapy can improve the prognosis of most locally advanced operable esophageal and gastric adenocarcinomas, but there are still some controversies about the best treatment [34].

We observed that nRT improved the OS of T3N0M0/T1-3N+M0 and T4 stage GEA patients. Especially for T4 patients, nRT has a significantly better impact on survival than aRT and surgery combined with chemotherapy. Most of the GEA patients with stage T4 invade adjacent structures (such as lung, large blood vessels, and trachea), and the prognosis is greatly dismal. Although modern surgical techniques have been significantly improved, these tumors are generally regarded as not directly surgically treated, which has also led to the increasingly prominent role of neoadjuvant therapy [35]. It is clear that patients with R0 resection have a longer survival period than R1 or R2 resection [36]. The nRT can transform unresectable or even inoperable tumors into resectable lesions, which cannot be achieved by postoperative adjuvant therapy. Analyses have shown that the median overall resection rate of T4 disease is 59% (35%-78%), and the R0 resection rate is 36.5% (32%-44%); this effect is mainly due to the role of neoadjuvant chemoradiotherapy [37]. Such achievement should enable patients with T4 stage esophagus or GEA without metastasis to be completely cured after R0 resection, thereby prolonging survival [38]. Therefore, a combination of nRT is likely the best choice when deciding the optimal scheme for treating patients with T4 GEA. Yet, the use of nRT in T4 patients is not ideal (only 28.63%) according to data extracted from the SEER database. Hence, the clinical importance of nRT for T4 GEA patients cannot be overemphasized.

Although we have extracted a large number of patient data with follow-up information from the SEER database, some of the inherent limitations of the database are related to the current research. However, as a national database, the SEER database does not provide information about the specific plan, dose, and duration of radiotherapy and chemotherapy. We can only determine whether the patient receives radiotherapy or chemotherapy and the sequence of radiotherapy and surgery. Among them, the information of radiation dose is particularly important. For example, a crossover test has shown that preoperative radiotherapy has survival benefits for GEA patients, but the dose of radiotherapy used is much lower than the dose often used in conventional neoadjuvant radiotherapy. Toxic and side effects caused by radiotherapy are an important issue that cannot be ignored in clinical practice. As the radiation dose increases, the toxicity may further increase. Different medical institutions in the United States have different radiation doses and techniques used in preoperative radiotherapy, which is a difference that cannot be balanced by the use of PSM in this study.

Another shortcoming is that there is no information on the patient’s tumor regression after radiotherapy, which has a great impact on the patient’s follow-up treatment and long-term survival. The SEER database, despite this limitation, is still a valuable database for studying cancer treatment. In addition, this study, as a retrospective analysis, perform propensity score matching to reduce some defects such as selection bias, but the conclusions should be ulteriorly proved by randomized controlled trials.

## Conclusions

In these carefully selected patients, the present study made the following recommendations: nRT can improve the prognosis of patients with T3N0M0/T1-3N+M0 and T4 Siewert type II GEA, and it seems to be a better treatment for T4 patients. Surgery alone seems to be sufficient, and nRT is not conducive to prolonging the survival of Siewert II GEA patients with T1-2N0M0 stage. Of course, further prospective trials are needed to verify this conclusion.

## Supporting information

S1 Table Features of stage T1-2N0M0 patients in the surgery only group and the neoadjuvant radiotherapy group before and after PSM. (DOCX)

S2 Table Features of stage T1-2N0M0 patients in the surgery plus chemotherapy group and the neoadjuvant radiotherapy group before and after PSM. (DOCX)

S3 Table Features of stage T1-2N0M0 patients in the adjuvant radiotherapy group and the neoadjuvant radiotherapy group before and after PSM. (DOCX)

S4 Table Features of stage T3N0M0/T1-3N+M0 patients in the surgery only group and the neoadjuvant radiotherapy group before and after PSM. (DOCX)

S5 Table Features of stage T3N0M0/T1-3N+M0 patients in the surgery plus chemotherapy group and the neoadjuvant radiotherapy group before and after PSM. (DOCX)

S6 Table Features of stage T3N0M0/T1-3N+M0 patients in the adjuvant radiotherapy group and the neoadjuvant radiotherapy group before and after PSM. (DOCX)

S7 Table Features of stage T4 patients in the surgery only group and the neoadjuvant radiotherapy group before and after PSM. (DOCX)

S8 Table Features of stage T4 patients in the surgery plus chemotherapy group and the neoadjuvant radiotherapy group before and after PSM. (DOCX)

S9 Table Features of stage T4 patients in the adjuvant radiotherapy group and the neoadjuvant radiotherapy group before and after PSM. (DOCX)

## Abbreviations

GEA: Gastroesophageal junction adenocarcinoma
GEJ: Gastroesophageal junction
SEER: Surveillance, Epidemiology, and End Results
nRT: Neoadjuvant radiotherapy
aRT: Adjuvant radiotherapy
PSM: Propensity score matching
OS: Overall survival
K-M: Kaplan-Meier
HR: Hazard ratio
RNE: Regional nodes examined
CI: Confidence interval
