# Supplementary material for: Neoadjuvant radiotherapy for locoregional Siewert type II gastroesophageal junction adenocarcinoma: A propensity scores matching analysis
Source: PLoS One. 2021 May 12;16(5):e0251555. doi: 10.1371/journal.pone.0251555 (PMC8115852; doi:10.1371/journal.pone.0251555)
Supplement: S1 Table — (DOCX) [file pone.0251555.s001.docx]

Supplementary Table 1. Features of stage T1-2N0M0 patients in the surgery only group and the neoadjuvant radiotherapy group before and after PSM.

| Characteristics | Before PSM | | |  | After PSM | | |
| --- | --- | --- | --- | --- | --- | --- | --- |
|  | Srugery only | Neoadjuvant radiotherapy | P |  | Srugery only | Neoadjuvant radiotherapy | P |
| Insurance Recode |  |  | 0.295 |  |  |  | 1.000 |
| No/Unknown | 523(30.39%) | 73(27.24%) |  |  | 67(26.59%) | 67(26.59%) |  |
| Insured | 1198(69.61%) | 195(72.76%) |  |  | 185(73.41%) | 185(73.41%) |  |
| Marital status |  |  | 0.094 |  |  |  | 1.000 |
| Single/Unknown | 577(33.53%) | 76(28.36%) |  |  | 69(27.38%) | 69(27.38%) |  |
| Married | 1144(66.47%) | 192(71.64%) |  |  | 183(72.62%) | 183(72.62%) |  |
| Race |  |  | 0.036 |  |  |  | 0.375 |
| Non-whites | 172(9.99%) | 16(5.97%) |  |  | 20(7.94%) | 14(5.56%) |  |
| White | 1549(90.01%) | 252(90.03%) |  |  | 232(92.06%) | 238(94.44%) |  |
| Age |  |  | <0.001 |  |  |  | 1.000 |
| <60 | 613(35.62%) | 131(48.88%) |  |  | 118(46.83%) | 118(46.83%) |  |
| ≥60 | 1108(64.38%) | 137(51.12%) |  |  | 134(53.17%) | 134(53.17%) |  |
| Sex |  |  | <0.001 |  |  |  | 0.522 |
| Female | 422(24.52%) | 39(14.55%) |  |  | 38(15.08%) | 33(13.10%) |  |
| Male | 1299(75.48%) | 229(85.45%) |  |  | 214(84.92%) | 219(86.90%) |  |
| Histology |  |  | 0.005 |  |  |  | 1.000 |
| Adenocarcinomas | 1610(93.55%) | 237(88.43%) |  |  | 224(88.89%) | 224(88.89%) |  |
| Cystic, mucinous and serous neoplasms | 111(6.45%) | 31(11.57%) |  |  | 28(11.11%) | 28(11.11%) |  |
| Grade |  |  | <0.001 |  |  |  | 0.070 |
| I | 272(15.80%) | 16(5.97%) |  |  | 22(8.73%) | 15(5.95%) |  |
| II | 725(42.13%) | 112(41.79%) |  |  | 111(44.05%) | 108(42.86%) |  |
| III/IV | 447(25.97%) | 103(38.43%) |  |  | 101(40.08%) | 94(37.30%) |  |
| Unknown | 277(16.10%) | 37(13.81%) |  |  | 18(7.14%) | 35(13.89%) |  |
| T stage |  |  | <0.001 |  |  |  | 1.000 |
| T1 | 1480(86.00%) | 134(50.00%) |  |  | 131(51.98%) | 131(51.98%) |  |
| T2 | 241(14.00%) | 134(50.00%) |  |  | 121(48.02%) | 121(48.02%) |  |
| RNE |  |  | 0.005 |  |  |  | 1.000 |
| <15 | 1219(70.83%) | 163(60.82%) |  |  | 154(61.11%) | 154(61.11%) |  |
| ≥15 | 477(27.72%) | 99(36.94%) |  |  | 94(37.30%) | 94(37.30%) |  |
| Unknown | 25(1.45%) | 6(2.24%) |  |  | 4(1.59%) | 4(1.59%) |  |
| Tumor size |  |  | <0.001 |  |  |  | 0.683 |
| <3cm | 756(43.93%) | 60(22.39%) |  |  | 64(25.40%) | 56(22.22%) |  |
| ≥3cm and <5cm | 501(29.11%) | 91(33.96%) |  |  | 91(36.11%) | 86(34.13%) |  |
| ≥5cm | 70(4.07%) | 32(11.94%) |  |  | 26(10.32%) | 29(11.51%) |  |
| Unknown | 394(22.89%) | 85(31.71%) |  |  | 71(28.17%) | 81(32.14%) |  |

Abbreviations PSM: Propensity score matching; RNE: Regional nodes examined
