# Supplementary material for: Neoadjuvant radiotherapy for locoregional Siewert type II gastroesophageal junction adenocarcinoma: A propensity scores matching analysis
Source: PLoS One. 2021 May 12;16(5):e0251555. doi: 10.1371/journal.pone.0251555 (PMC8115852; doi:10.1371/journal.pone.0251555)
Supplement: S2 Table — (DOCX) [file pone.0251555.s002.docx]

Supplementary Table 2. Features of stage T1-2N0M0 patients in the surgery plus chemotherapy group and the neoadjuvant radiotherapy group before and after PSM.

| Characteristics | Before PSM | | |  | After PSM | | |
| --- | --- | --- | --- | --- | --- | --- | --- |
|  | surgery plus chemotherapy | Neoadjuvant radiotherapy | P |  | surgery plus chemotherapy | Neoadjuvant radiotherapy | P |
| Insurance Recode |  |  | 0.868 |  |  |  | 0.563 |
| No/Unknown | 32(28.07%) | 73(27.24%) |  |  | 32(28.07%) | 36(31.58%) |  |
| Insured | 82(71.93%) | 195(72.76%) |  |  | 82(71.93%) | 78(68.42%) |  |
| Marital status |  |  | 0.254 |  |  |  | 0.391 |
| Single/Unknown | 39(34.22%) | 76(28.36%) |  |  | 39(34.22%) | 32(28.07%) |  |
| Married | 75(65.78%) | 192(71.64%) |  |  | 75(65.78%) | 82(71.93%) |  |
| Race |  |  | 0.019 |  |  |  | 0.397 |
| Non-whites | 15(13.16%) | 16(5.97%) |  |  | 15(13.16%) | 10(8.77%) |  |
| White | 99(86.84%) | 252(90.03%) |  |  | 99(86.84%) | 104(91.23%) |  |
| Age |  |  | 0.787 |  |  |  | 1.000 |
| <60 | 54(47.37%) | 131(48.88%) |  |  | 54(47.37%) | 54(47.37%) |  |
| ≥60 | 60(52.63%) | 137(51.12%) |  |  | 60(52.63%) | 60(52.63%) |  |
| Sex |  |  | 0.054 |  |  |  | 0.128 |
| Female | 26(22.81%) | 39(14.55%) |  |  | 26(22.81%) | 17(14.91%) |  |
| Male | 88(77.19%) | 229(85.45%) |  |  | 88(77.19%) | 97(85.09%) |  |
| Histology |  |  | 0.768 |  |  |  | 1.000 |
| Adenocarcinomas | 102(89.47%) | 237(88.43%) |  |  | 102(89.47%) | 102(89.47%) |  |
| Cystic, mucinous and serous neoplasms | 12(10.53%) | 31(11.57%) |  |  | 12(10.53%) | 12(10.53%) |  |
| Grade |  |  | 0.914 |  |  |  | 0.831 |
| I | 7(6.14%) | 16(5.97%) |  |  | 7(6.14%) | 5(4.39%) |  |
| II | 45(39.47%) | 112(41.79%) |  |  | 45(39.47%) | 43(37.72%) |  |
| III/IV | 48(42.11%) | 103(38.43%) |  |  | 48(42.11%) | 48(42.11%) |  |
| Unknown | 14(12.28%) | 37(13.81%) |  |  | 14(12.28%) | 18(15.78%) |  |
| T stage |  |  | 0.018 |  |  |  | 0.082 |
| T1 | 72(63.16%) | 134(50.00%) |  |  | 72(63.16%) | 59(51.75%) |  |
| T2 | 42(36.84%) | 134(50.00%) |  |  | 42(36.84%) | 55(48.25%) |  |
| RNE |  |  | 0.282 |  |  |  | 0.965 |
| <15 | 60(52.63%) | 163(60.82%) |  |  | 60(52.63%) | 62(54.39%) |  |
| ≥15 | 52(45.62%) | 99(36.94%) |  |  | 52(45.62%) | 50(43.86%) |  |
| Unknown | 2(1.75%) | 6(2.24%) |  |  | 2(1.75%) | 2(1.75%) |  |
| Tumor size |  |  | 0.434 |  |  |  | 0.338 |
| <3cm | 27(23.68%) | 60(22.39%) |  |  | 27(23.68%) | 27(23.68%) |  |
| ≥3cm and <5cm | 47(41.23%) | 91(33.96%) |  |  | 47(41.23%) | 35(30.70%) |  |
| ≥5cm | 10(8.77%) | 32(11.94%) |  |  | 10(8.77%) | 12(10.53%) |  |
| Unknown | 30(26.32%) | 85(31.71%) |  |  | 30(26.32%) | 40(35.09%) |  |

Abbreviations PSM: Propensity score matching; RNE: Regional nodes examined
