# Supplementary material for: Neoadjuvant radiotherapy for locoregional Siewert type II gastroesophageal junction adenocarcinoma: A propensity scores matching analysis
Source: PLoS One. 2021 May 12;16(5):e0251555. doi: 10.1371/journal.pone.0251555 (PMC8115852; doi:10.1371/journal.pone.0251555)
Supplement: S3 Table — (DOCX) [file pone.0251555.s003.docx]

Supplementary Table 3. Features of stage T1-2N0M0 patients in the adjuvant radiotherapy group and the neoadjuvant radiotherapy group before and after PSM.

| Characteristics | Before PSM | | |  | After PSM | | |
| --- | --- | --- | --- | --- | --- | --- | --- |
|  | Adjuvant radiotherapy | Neoadjuvant radiotherapy | P |  | Adjuvant radiotherapy | Neoadjuvant radiotherapy | P |
| Insurance Recode |  |  | 0.640 |  |  |  | 0.659 |
| No/Unknown | 32(29.63%) | 73(27.24%) |  |  | 32(29.63%) | 35(32.41%) |  |
| Insured | 76(70.37%) | 195(72.76%) |  |  | 76(70.37%) | 73(67.59%) |  |
| Marital status |  |  | 0.193 |  |  |  | 0.469 |
| Single/Unknown | 38(35.19%) | 76(28.36%) |  |  | 38(35.19%) | 33(30.56%) |  |
| Married | 70(64.81%) | 192(71.64%) |  |  | 70(64.81%) | 75(69.44%) |  |
| Race |  |  | 0.005 |  |  |  | 0.252 |
| Non-whites | 9(8.33%) | 16(5.97%) |  |  | 9(8.33%) | 4(3.70%) |  |
| White | 99(91.67%) | 252(90.03%) |  |  | 99(91.67%) | 104(96.30%) |  |
| Age |  |  | 0.006 |  |  |  | 1.000 |
| <60 | 36(33.33%) | 131(48.88%) |  |  | 36(33.33%) | 36(33.33%) |  |
| ≥60 | 72(66.67%) | 137(51.12%) |  |  | 72(66.67%) | 72(66.67%) |  |
| Sex |  |  | 0.166 |  |  |  | 0.201 |
| Female | 22(20.37%) | 39(14.55%) |  |  | 22(20.37%) | 14(12.96%) |  |
| Male | 86(76.63%) | 229(85.45%) |  |  | 86(76.63%) | 94(87.04%) |  |
| Histology |  |  | 0.008 |  |  |  | 0.280 |
| Adenocarcinomas | 106(98.15%) | 237(88.43%) |  |  | 106(98.15%) | 102(94.44%) |  |
| Cystic, mucinous and serous neoplasms | 2(1.85%) | 31(11.57%) |  |  | 2(1.85%) | 6(5.56%) |  |
| Grade |  |  | 0.072 |  |  |  | 0.074 |
| I | 15(13.89%) | 16(5.97%) |  |  | 15(13.89%) | 6(5.56%) |  |
| II | 45(41.67%) | 112(41.79%) |  |  | 45(41.67%) | 38(35.18%) |  |
| III/IV | 37(34.26%) | 103(38.43%) |  |  | 37(34.26%) | 47(43.52%) |  |
| Unknown | 11(10.18%) | 37(13.81%) |  |  | 11(10.18%) | 17(15.74%) |  |
| T stage |  |  | 0.002 |  |  |  | 0.069 |
| T1 | 73(67.59%) | 134(50.00%) |  |  | 73(67.59%) | 60(55.56%) |  |
| T2 | 35(32.41%) | 134(50.00%) |  |  | 35(32.41%) | 48(44.44%) |  |
| RNE |  |  | <0.001 |  |  |  | 0.897 |
| <15 | 94(87.04%) | 163(60.82%) |  |  | 94(87.04%) | 96(88.89%) |  |
| ≥15 | 10(9.26%) | 99(36.94%) |  |  | 10(9.26%) | 9(8.33%) |  |
| Unknown | 4(3.70%) | 6(2.24%) |  |  | 4(3.70%) | 3(2.78%) |  |
| Tumor size |  |  | 0.029 |  |  |  | 0.207 |
| <3cm | 40(37.04%) | 60(22.39%) |  |  | 40(37.04%) | 26(24.07%) |  |
| ≥3cm and <5cm | 30(27.78%) | 91(33.96%) |  |  | 30(27.78%) | 34(31.48%) |  |
| ≥5cm | 8(7.40%) | 32(11.94%) |  |  | 8(7.40%) | 12(11.11%) |  |
| Unknown | 30(27.78%) | 85(31.71%) |  |  | 30(27.78%) | 36(33.34%) |  |

Abbreviations PSM: Propensity score matching; RNE: Regional nodes examined
