# Supplementary material for: Neoadjuvant radiotherapy for locoregional Siewert type II gastroesophageal junction adenocarcinoma: A propensity scores matching analysis
Source: PLoS One. 2021 May 12;16(5):e0251555. doi: 10.1371/journal.pone.0251555 (PMC8115852; doi:10.1371/journal.pone.0251555)
Supplement: S4 Table — (DOCX) [file pone.0251555.s004.docx]

Supplementary Table 4. Features of stage T3N0M0/T1-3N+M0 patients in the surgery only group and the neoadjuvant radiotherapy group before and after PSM.

| Characteristics | Before PSM | | |  | After PSM | | |
| --- | --- | --- | --- | --- | --- | --- | --- |
|  | Srugery only | Neoadjuvant radiotherapy | P |  | Srugery only | Neoadjuvant radiotherapy | P |
| Insurance Recode |  |  | <0.001 |  |  |  | 0.161 |
| No/Unknown | 80(33.33%) | 87(18.51%) |  |  | 79(33.76%) | 65(27.78%) |  |
| Insured | 160(66.67%) | 383(81.49%) |  |  | 155(66.24%) | 169(72.22%) |  |
| Marital status |  |  | 0.051 |  |  |  | 0.174 |
| Single/Unknown | 91(37.92%) | 144(30.64%) |  |  | 88(37.61%) | 74(31.62%) |  |
| Married | 149(62.08%) | 326(69.36%) |  |  | 146(62.39%) | 160(68.38%) |  |
| Race |  |  | 0.006 |  |  |  | 0.386 |
| Non-whites | 34(14.17%) | 36(7.66%) |  |  | 32(13.68%) | 17(7.26%) |  |
| White | 206(85.83%) | 434(92.34%) |  |  | 202(86.32%) | 217(92.74%) |  |
| Age |  |  | <0.001 |  |  |  | 1.000 |
| <60 | 56(23.33%) | 247(52.55%) |  |  | 56(23.93%) | 56(23.93%) |  |
| ≥60 | 184(76.67%) | 223(47.45%) |  |  | 178(76.07%) | 178(76.07%) |  |
| Sex |  |  | 0.002 |  |  |  | 0.125 |
| Female | 63(26.25%) | 77(16.38%) |  |  | 61(26.07%) | 47(20.09%) |  |
| Male | 177(73.75%) | 393(83.62%) |  |  | 173(73.93%) | 187(79.91%) |  |
| Histology |  |  | 0.198 |  |  |  | 0.128 |
| Adenocarcinomas | 209(87.08%) | 392(83.40%) |  |  | 203(86.75%) | 191(81.62%) |  |
| Cystic, mucinous and serous neoplasms | 31(12.92%) | 78(16.60%) |  |  | 31(13.25%) | 43(18.38%) |  |
| Grade |  |  | <0.001 |  |  |  | 0.544 |
| I | 11(4.58%) | 30(6.38%) |  |  | 11(4.70%) | 17(7.26%) |  |
| II | 97(40.42%) | 153(32.55%) |  |  | 94(40.17%) | 94(40.17%) |  |
| III/IV | 125(52.08%) | 227(48.30%) |  |  | 122(52.14%) | 119(50.86%) |  |
| Unknown | 7(2.92%) | 60(12.77%) |  |  | 7(2.99%) | 4(1.71%) |  |
| T stage |  |  | 0.421 |  |  |  | 1.000 |
| T1 | 1 (0.42%) | 7(1.49%) |  |  | 1(0.43%) | 1(0.43%) |  |
| T2 | 6(2.50%) | 10(2.13%) |  |  | 4(1.71%) | 4(1.71%) |  |
| T3 | 233(97.08%) | 453(96.38%) |  |  | 229(97.86%) | 229(97.86%) |  |
| N stage |  |  | 0.002 |  |  |  | 1.000 |
| N0 | 207(86.25%) | 369(78.51%) |  |  | 207(88.46%) | 207(88.46%) |  |
| N1 | 15(6.25%) | 50(10.64%) |  |  | 13(5.56%) | 13(5.56%) |  |
| N2 | 8(3.33%) | 42(8.94%) |  |  | 7(2.99%) | 7(2.99%) |  |
| N3 | 10(4.17%) | 9(1.91%) |  |  | 7(2.99%) | 7(2.99%) |  |
| RNE |  |  | 0.327 |  |  |  | 1.000 |
| <15 | 126(52.50%) | 266(56.60%) |  |  | 123(52.56%) | 123(52.56%) |  |
| ≥15 | 112(46.67%) | 196(41.70%) |  |  | 110(47.01%) | 110(47.01%) |  |
| Unknown | 2(0.83%) | 8(1.70%) |  |  | 1(0.43%) | 1(0.43%) |  |
| Tumor size |  |  | <0.001 |  |  |  | 0.319 |
| <3cm | 18(7.50%) | 49(10.43%) |  |  | 18(7.69%) | 28(11.97%) |  |
| ≥3cm and <5cm | 115(47.92%) | 208(44.26%) |  |  | 112(47.86%) | 99(42.31%) |  |
| ≥5cm | 94(39.17%) | 116(24.68%) |  |  | 91(38.89%) | 90(38.46%) |  |
| Unknown | 13(5.41%) | 97(20.63%) |  |  | 13(5.56%) | 17(7.26%) |  |

Abbreviations PSM: Propensity score matching; RNE: Regional nodes examined
