# Supplementary material for: Neoadjuvant radiotherapy for locoregional Siewert type II gastroesophageal junction adenocarcinoma: A propensity scores matching analysis
Source: PLoS One. 2021 May 12;16(5):e0251555. doi: 10.1371/journal.pone.0251555 (PMC8115852; doi:10.1371/journal.pone.0251555)
Supplement: S5 Table — (DOCX) [file pone.0251555.s005.docx]

Supplementary Table 5. Features of stage T3N0M0/T1-3N+M0 patients in the surgery plus chemotherapy group and the neoadjuvant radiotherapy group before and after PSM.

| Characteristics | Before PSM | | |  | After PSM | | |
| --- | --- | --- | --- | --- | --- | --- | --- |
|  | Srugery plus chemotherapy | Neoadjuvant radiotherapy | P |  | Srugery plus chemotherapy | Neoadjuvant radiotherapy | P |
| Insurance Recode |  |  | 0.752 |  |  |  | 0.076 |
| No/Unknown | 27(19.71%) | 87(18.51%) |  |  | 24(19.35%) | 37(29.84%) |  |
| Insured | 110(80.29%) | 383(81.49%) |  |  | 100(80.65%) | 87(70.16%) |  |
| Marital status |  |  | 0.623 |  |  |  | 0.259 |
| Single/Unknown | 45(32.85%) | 144(30.64%) |  |  | 39(31.45%) | 31(25.00%) |  |
| Married | 92(67.15%) | 326(69.36%) |  |  | 85(68.55%) | 93(75.00%) |  |
| Race |  |  | 0.338 |  |  |  | 0.535 |
| Non-whites | 14(10.22%) | 36(7.66%) |  |  | 11(8.87%) | 15(12.10%) |  |
| White | 123(89.78%) | 434(92.34%) |  |  | 113(91.13%) | 109(87.90%) |  |
| Age |  |  | 0.071 |  |  |  | 1.000 |
| <60 | 60(43.80%) | 247(52.55%) |  |  | 56(45.16%) | 56(45.16%) |  |
| ≥60 | 77(56.20%) | 223(47.45%) |  |  | 68(54.84%) | 68(54.84%) |  |
| Sex |  |  | 0.768 |  |  |  | 0.866 |
| Female | 21(15.33%) | 77(16.38%) |  |  | 20(16.13%) | 22(17.74%) |  |
| Male | 116(84.67%) | 393(83.62%) |  |  | 104(83.87%) | 102(82.26%) |  |
| Histology |  |  | 0.397 |  |  |  | 1.000 |
| Adenocarcinomas | 110(80.29%) | 392(83.40%) |  |  | 101(81.45%) | 101(81.45%) |  |
| Cystic, mucinous and serous neoplasms | 27(19.71%) | 78(16.60%) |  |  | 23(18.55%) | 23(18.55%) |  |
| Grade |  |  | 0.001 |  |  |  | 0.207 |
| I | 6(4.38%) | 30(6.38%) |  |  | 5(4.03%) | 10(8.06%) |  |
| II | 36(26.28%) | 153(32.55%) |  |  | 34(27.42%) | 41(33.06%) |  |
| III/IV | 90(65.69%) | 227(48.30%) |  |  | 80(64.52%) | 65(52.42%) |  |
| Unknown | 5(3.65%) | 60(12.77%) |  |  | 5(4.03%) | 8(6.46%) |  |
| T stage |  |  | 0.686 |  |  |  | 1.000 |
| T1 | 1 (0.73%) | 7(1.49%) |  |  | 1(0.81%) | 1(0.81%) |  |
| T2 | 4(2.92%) | 10(2.13%) |  |  | 3(2.42%) | 3(2.42%) |  |
| T3 | 132(96.35%) | 453(96.38%) |  |  | 120(96.77%) | 120(96.77%) |  |
| N stage |  |  | <0.001 |  |  |  | 1.000 |
| N0 | 95(69.34%) | 369(78.51%) |  |  | 95(76.61%) | 95(76.61%) |  |
| N1 | 17(12.41%) | 50(10.64%) |  |  | 15(12.10%) | 15(12.10%) |  |
| N2 | 11(8.03%) | 42(8.94%) |  |  | 10(8.06%) | 10(8.06%) |  |
| N3 | 14(10.22%) | 9(1.91%) |  |  | 4(3.23%) | 4(3.23%) |  |
| RNE |  |  | 0.008 |  |  |  | 1.000 |
| <15 | 57(41.61%) | 266(56.60%) |  |  | 53(42.74%) | 53(42.74%) |  |
| ≥15 | 76(55.47%) | 196(41.70%) |  |  | 68(54.84%) | 68(54.84%) |  |
| Unknown | 4(2.92%) | 8(1.70%) |  |  | 3(2.42%) | 3(2.42%) |  |
| Tumor size |  |  | 0.400 |  |  |  | 0.843 |
| <3cm | 12(8.76%) | 49(10.43%) |  |  | 12(9.68%) | 13(10.48%) |  |
| ≥3cm and <5cm | 59(43.07%) | 208(44.26%) |  |  | 53(42.74%) | 46(37.10%) |  |
| ≥5cm | 43(31.39%) | 116(24.68%) |  |  | 36(29.03%) | 40(32.26%) |  |
| Unknown | 23(16.78%) | 97(20.63%) |  |  | 23(18.55%) | 25(20.16%) |  |

Abbreviations PSM: Propensity score matching; RNE: Regional nodes examined
