# Supplementary material for: Neoadjuvant radiotherapy for locoregional Siewert type II gastroesophageal junction adenocarcinoma: A propensity scores matching analysis
Source: PLoS One. 2021 May 12;16(5):e0251555. doi: 10.1371/journal.pone.0251555 (PMC8115852; doi:10.1371/journal.pone.0251555)
Supplement: S6 Table — (DOCX) [file pone.0251555.s006.docx]

Supplementary Table 6. Features of stage T3N0M0/T1-3N+M0 patients in the adjuvant radiotherapy group and the neoadjuvant radiotherapy group before and after PSM.

| Characteristics | Before PSM | | |  | After PSM | | |
| --- | --- | --- | --- | --- | --- | --- | --- |
|  | Adjuvant radiotherapy | Neoadjuvant radiotherapy | P |  | Adjuvant radiotherapy | Neoadjuvant radiotherapy | P |
| Insurance Recode |  |  | 0.283 |  |  |  | 1.000 |
| No/Unknown | 27(22.88%) | 87(18.51%) |  |  | 22(20.56%) | 22(20.56%) |  |
| Insured | 91(77.12%) | 383(81.49%) |  |  | 85(79.44%) | 85(79.44%) |  |
| Marital status |  |  | 0.837 |  |  |  | 0.354 |
| Single/Unknown | 35(29.66%) | 144(30.64%) |  |  | 32(29.91%) | 25(23.36%) |  |
| Married | 83(70.34%) | 326(69.36%) |  |  | 75(70.09%) | 82(76.64%) |  |
| Race |  |  | 0.008 |  |  |  | 0.087 |
| Non-whites | 19(16.10%) | 36(7.66%) |  |  | 17(15.89%) | 8(7.48%) |  |
| White | 99(83.90%) | 434(92.34%) |  |  | 90(84.11%) | 99(92.52%) |  |
| Age |  |  | 0.743 |  |  |  | 0.784 |
| <60 | 64(54.24%) | 247(52.55%) |  |  | 57(53.27%) | 55(51.40%) |  |
| ≥60 | 54(45.76%) | 223(47.45%) |  |  | 50(46.73%) | 52(48.60%) |  |
| Sex |  |  | 0.218 |  |  |  | 0.479 |
| Female | 25(21.19%) | 77(16.38%) |  |  | 22(20.56%) | 17(15.89%) |  |
| Male | 93(78.81%) | 393(83.62%) |  |  | 85(79.44%) | 90(84.11%) |  |
| Histology |  |  | 0.241 |  |  |  | 0.733 |
| Adenocarcinomas | 93(78.81%) | 392(83.40%) |  |  | 87(81.45%) | 84(81.45%) |  |
| Cystic, mucinous and serous neoplasms | 25(21.19%) | 78(16.60%) |  |  | 20(18.55%) | 23(18.55%) |  |
| Grade |  |  | 0.001 |  |  |  | 0.765 |
| I | 5(4.24%) | 30(6.38%) |  |  | 5(4.67%) | 8(7.48%) |  |
| II | 36(30.51%) | 153(32.55%) |  |  | 33(30.84%) | 29(27.10%) |  |
| III/IV | 75(63.56%) | 227(48.30%) |  |  | 67(62.62%) | 67(62.62%) |  |
| Unknown | 2(1.69%) | 60(12.77%) |  |  | 2(1.87%) | 3(2.80%) |  |
| T stage |  |  | 0.186 |  |  |  | 0.101 |
| T1 | 1 (0.85%) | 7(1.49%) |  |  | 1(0.93%) | 6(5.61%) |  |
| T2 | 6(5.08%) | 10(2.13%) |  |  | 5(4.67%) | 8(7.48%) |  |
| T3 | 111(94.07%) | 453(96.38%) |  |  | 101(94.40%) | 93(86.91%) |  |
| N stage |  |  | <0.001 |  |  |  | 1.000 |
| N0 | 74(62.71%) | 369(78.51%) |  |  | 74(69.16%) | 74(69.16%) |  |
| N1 | 22(18.64%) | 50(10.64%) |  |  | 19(17.76%) | 19(17.76%) |  |
| N2 | 9(7.63%) | 42(8.94%) |  |  | 7(6.54%) | 7(6.54%) |  |
| N3 | 13(11.02%) | 9(1.91%) |  |  | 7(6.54%) | 7(6.54%) |  |
| RNE |  |  | 0.896 |  |  |  | 0.704 |
| <15 | 64(54.24%) | 266(56.60%) |  |  | 56(52.34%) | 54(50.47%) |  |
| ≥15 | 52(44.07%) | 196(41.70%) |  |  | 49(45.79%) | 49(45.79%) |  |
| Unknown | 2(1.69%) | 8(1.70%) |  |  | 2(1.87%) | 4(3.74%) |  |
| Tumor size |  |  | <0.001 |  |  |  | 0.154 |
| <3cm | 3(2.54%) | 49(10.43%) |  |  | 3(2.80%) | 5(4.67%) |  |
| ≥3cm and <5cm | 54(45.76%) | 208(44.26%) |  |  | 50(46.73%) | 49(45.79%) |  |
| ≥5cm | 48(40.68%) | 116(24.68%) |  |  | 43(40.19%) | 32(29.91%) |  |
| Unknown | 13(11.02%) | 97(20.63%) |  |  | 11(10.28%) | 21(19.63%) |  |

Abbreviations PSM: Propensity score matching; RNE: Regional nodes examined
