# Supplementary material for: Neoadjuvant radiotherapy for locoregional Siewert type II gastroesophageal junction adenocarcinoma: A propensity scores matching analysis
Source: PLoS One. 2021 May 12;16(5):e0251555. doi: 10.1371/journal.pone.0251555 (PMC8115852; doi:10.1371/journal.pone.0251555)
Supplement: S7 Table — (DOCX) [file pone.0251555.s007.docx]

Supplementary Table 7. Features of stage T4 patients in the surgery only group and the neoadjuvant radiotherapy group before and after PSM.

| Characteristics | Before PSM | | |  | After PSM | | |
| --- | --- | --- | --- | --- | --- | --- | --- |
|  | Srugery only | Neoadjuvant radiotherapy | P |  | Srugery only | Neoadjuvant radiotherapy | P |
| Insurance Recode |  |  | <0.001 |  |  |  | 1.000 |
| No/Unknown | 141(60.00%) | 110(39.29%) |  |  | 53(55.21%) | 53(55.21%) |  |
| Insured | 94(40.00%) | 170(60.71%) |  |  | 43(44.79%) | 43(44.79%) |  |
| Marital status |  |  | <0.001 |  |  |  | 1.000 |
| Single/Unknown | 107(45.53%) | 77(27.50%) |  |  | 29(30.21%) | 29(30.21%) |  |
| Married | 128(54.47%) | 203(72.50%) |  |  | 67(69.79%) | 67(69.79%) |  |
| Race |  |  | <0.001 |  |  |  | 0.391 |
| Non-whites | 42(17.87%) | 17(6.07%) |  |  | 15(15.63%) | 10(10.42%) |  |
| White | 193(82.13%) | 263(93.93%) |  |  | 81(84.37%) | 86(89.58%) |  |
| Age |  |  | <0.001 |  |  |  | 1.000 |
| <60 | 70(29.79%) | 186(66.43%) |  |  | 46(47.92%) | 46(47.92%) |  |
| ≥60 | 165(70.21%) | 94(33.57%) |  |  | 50(52.08%) | 50(52.08%) |  |
| Sex |  |  | 0.001 |  |  |  | 0.066 |
| Female | 65(27.66%) | 45(16.07%) |  |  | 24(25.00%) | 13(13.54%) |  |
| Male | 170(72.34%) | 235(83.93%) |  |  | 72(75.00%) | 83(86.46%) |  |
| Histology |  |  | 0.066 |  |  |  | 0.687 |
| Adenocarcinomas | 189(80.43%) | 242(86.43%) |  |  | 80(83.33%) | 83(86.46%) |  |
| Cystic, mucinous and serous neoplasms | 46(19.57%) | 38(13.57%) |  |  | 16(16.67%) | 13(13.54%) |  |
| Grade |  |  | 0.006 |  |  |  | 1.000 |
| I | 7(2.98%) | 14(5.00%) |  |  | 2(2.08%) | 2(2.08%) |  |
| II | 58(24.68%) | 87(31.07%) |  |  | 25(26.04%) | 25(26.04%) |  |
| III/IV | 160(68.08%) | 152(54.29%) |  |  | 67(69.80%) | 67(69.80%) |  |
| Unknown | 10(4.26%) | 27(9.64%) |  |  | 2(2.08%) | 2(2.08%) |  |
| N stage |  |  | 0.312 |  |  |  | 1.000 |
| N0 | 53(22.55%) | 62(22.14%) |  |  | 23(23.96%) | 23(23.96%) |  |
| N1 | 7(2.98%) | 16(5.71%) |  |  | 1(1.04%) | 1(1.04%) |  |
| N2 | 1(0.43%) | 5(1.79%) |  |  | - | - |  |
| N3 | 3(1.28%) | 2(0.71%) |  |  | - | - |  |
| Nx | 171(72.76%) | 195(69.65%) |  |  | 72(75.00%) | 72(75.00%) |  |
| RNE |  |  | 0.327 |  |  |  | 0.140 |
| <15 | 136(57.87%) | 176(62.86%) |  |  | 53(55.21%) | 65(67.71%) |  |
| ≥15 | 96(40.85%) | 98(35.00%) |  |  | 42(43.75%) | 29(30.21%) |  |
| Unknown | 3(1.28%) | 6(2.14%) |  |  | 1(1.04%) | 2(2.08%) |  |
| Tumor size |  |  | <0.001 |  |  |  | 0.754 |
| <3cm | 6(2.55%) | 11(3.93%) |  |  | 2(2.08%) | 2(2.08%) |  |
| ≥3cm and <5cm | 94(40.00%) | 107(38.21%) |  |  | 40(41.67%) | 42(43.75%) |  |
| ≥5cm | 115(48.94%) | 88(31.43%) |  |  | 45(46.88%) | 47(48.96%) |  |
| Unknown | 20(8.51%) | 74(26.43%) |  |  | 9(9.37%) | 5(5.21%) |  |

Abbreviations PSM: Propensity score matching; RNE: Regional nodes examined
