# Supplementary material for: Neoadjuvant radiotherapy for locoregional Siewert type II gastroesophageal junction adenocarcinoma: A propensity scores matching analysis
Source: PLoS One. 2021 May 12;16(5):e0251555. doi: 10.1371/journal.pone.0251555 (PMC8115852; doi:10.1371/journal.pone.0251555)
Supplement: S8 Table — (DOCX) [file pone.0251555.s008.docx]

Supplementary Table 8. Features of stage T4 patients in the surgery plus chemotherapy group and the neoadjuvant radiotherapy group before and after PSM.

| Characteristics | Before PSM | | |  | After PSM | | |
| --- | --- | --- | --- | --- | --- | --- | --- |
|  | Srugery plus chemotherapy | Neoadjuvant radiotherapy | P |  | Srugery plus chemotherapy | Neoadjuvant radiotherapy | P |
| Insurance Recode |  |  | 0.327 |  |  |  | 1.000 |
| No/Unknown | 79(43.89%) | 110(39.29%) |  |  | 63(41.45%) | 63(41.45%) |  |
| Insured | 101(56.11%) | 170(60.71%) |  |  | 89(58.55%) | 89(58.55%) |  |
| Marital status |  |  | 0.480 |  |  |  | 0.697 |
| Single/Unknown | 55(30.56%) | 77(27.50%) |  |  | 42(27.63%) | 39(25.66%) |  |
| Married | 125(69.44%) | 203(72.50%) |  |  | 110(72.37%) | 113(74.34%) |  |
| Race |  |  | 0.001 |  |  |  | 0.396 |
| Non-whites | 29(16.11%) | 17(6.07%) |  |  | 23(15.13%) | 17(11.18%) |  |
| White | 151(83.89%) | 263(93.93%) |  |  | 129(84.87%) | 135(88.82%) |  |
| Age |  |  | 0.161 |  |  |  | 1.000 |
| <60 | 108(60.00%) | 186(66.43%) |  |  | 96(63.16%) | 96(63.16%) |  |
| ≥60 | 72(40.00%) | 94(33.57%) |  |  | 56(36.84%) | 56(36.84%) |  |
| Sex |  |  | 0.170 |  |  |  | 0.365 |
| Female | 38(21.11%) | 45(16.07%) |  |  | 30(19.74%) | 23(15.13%) |  |
| Male | 142(78.89%) | 235(83.93%) |  |  | 122(80.26%) | 129(84.87%) |  |
| Histology |  |  | 0.002 |  |  |  | 1.000 |
| Adenocarcinomas | 135(75.00%) | 242(86.43%) |  |  | 126(82.89%) | 126(82.89%) |  |
| Cystic, mucinous and serous neoplasms | 45(25.00%) | 38(13.57%) |  |  | 26(17.11%) | 26(17.11%) |  |
| Grade |  |  | 0.002 |  |  |  | 1.000 |
| I | 3(1.67%) | 14(5.00%) |  |  | 3(1.97%) | 3(1.97%) |  |
| II | 41(22.78%) | 87(31.07%) |  |  | 37(24.34%) | 37(24.34%) |  |
| III/IV | 128(71.11%) | 152(54.29%) |  |  | 107(70.39%) | 107(70.39%) |  |
| Unknown | 8(4.44%) | 27(9.64%) |  |  | 5(3.30%) | 5(3.30%) |  |
| N stage |  |  | 0.061 |  |  |  | 1.000 |
| N0 | 26(14.44%) | 62(22.14%) |  |  | 22(14.47%) | 22(14.47%) |  |
| N1 | 6(3.33%) | 16(5.71%) |  |  | 5(3.29%) | 5(3.29%) |  |
| N2 | 5(2.78%) | 5(1.79%) |  |  | 1(0.66%) | 1(0.66%) |  |
| N3 | 5(2.78%) | 2(0.71%) |  |  | - | - |  |
| Nx | 138(76.67%) | 195(69.65%) |  |  | 124(81.58%) | 124(81.58%) |  |
| RNE |  |  | <0.001 |  |  |  | 0.749 |
| <15 | 65(36.11%) | 176(62.86%) |  |  | 59(38.82%) | 65(42.76%) |  |
| ≥15 | 110(61.11%) | 98(35.00%) |  |  | 89(58.55%) | 84(55.26%) |  |
| Unknown | 5(2.78%) | 6(2.14%) |  |  | 4(2.63%) | 3(1.98%) |  |
| Tumor size |  |  | 0.004 |  |  |  | 0.318 |
| <3cm | 5(2.78%) | 11(3.93%) |  |  | 5(3.29%) | 3(1.97%) |  |
| ≥3cm and <5cm | 53(29.44%) | 107(38.21%) |  |  | 46(30.26%) | 59(38.82%) |  |
| ≥5cm | 87(48.33%) | 88(31.43%) |  |  | 72(47.37%) | 51(33.55%) |  |
| Unknown | 35(19.45%) | 74(26.43%) |  |  | 29(19.08%) | 39(25.66%) |  |

Abbreviations PSM: Propensity score matching; RNE: Regional nodes examined
