# Supplementary material for: Neoadjuvant radiotherapy for locoregional Siewert type II gastroesophageal junction adenocarcinoma: A propensity scores matching analysis
Source: PLoS One. 2021 May 12;16(5):e0251555. doi: 10.1371/journal.pone.0251555 (PMC8115852; doi:10.1371/journal.pone.0251555)
Supplement: S9 Table — (DOCX) [file pone.0251555.s009.docx]

Supplementary Table 9. Features of stage T4 patients in the adjuvant radiotherapy group and the neoadjuvant radiotherapy group before and after PSM.

| Characteristics | Before PSM | | |  | After PSM | | |
| --- | --- | --- | --- | --- | --- | --- | --- |
|  | Adjuvant radiotherapy | Neoadjuvant radiotherapy | P |  | Adjuvant radiotherapy | Neoadjuvant radiotherapy | P |
| Insurance Recode |  |  | 0.002 |  |  |  | 0.418 |
| No/Unknown | 147(52.50%) | 110(39.29%) |  |  | 119(47.79%) | 110(41.45%) |  |
| Insured | 133(47.50%) | 170(60.71%) |  |  | 130(52.21%) | 139(58.55%) |  |
| Marital status |  |  | 0.037 |  |  |  | 0.052 |
| Single/Unknown | 100(35.71%) | 77(27.50%) |  |  | 87(34.94%) | 66(26.51%) |  |
| Married | 180(64.29%) | 203(72.50%) |  |  | 162(65.06%) | 183(73.49%) |  |
| Race |  |  | 0.001 |  |  |  | 1.000 |
| Non-whites | 42(15.00%) | 17(6.07%) |  |  | 15(6.02%) | 16(6.42%) |  |
| White | 238(85.00%) | 263(93.93%) |  |  | 234(93.98%) | 233(93.58%) |  |
| Age |  |  | 0.096 |  |  |  | 1.000 |
| <60 | 167(59.64%) | 186(66.43%) |  |  | 159(63.86%) | 159(63.86%) |  |
| ≥60 | 113(40.36%) | 94(33.57%) |  |  | 90(36.14%) | 90(36.14%) |  |
| Sex |  |  | 0.012 |  |  |  | 0.459 |
| Female | 69(24.64%) | 45(16.07%) |  |  | 42(16.87%) | 36(14.46%) |  |
| Male | 211(75.36%) | 235(83.93%) |  |  | 207(83.13%) | 213(85.54%) |  |
| Histology |  |  | 0.004 |  |  |  | 0.111 |
| Adenocarcinomas | 216(77.14%) | 242(86.43%) |  |  | 203(81.53%) | 216(86.75%) |  |
| Cystic, mucinous and serous neoplasms | 64(22.86%) | 38(13.57%) |  |  | 46(18.47%) | 33(13.25%) |  |
| Grade |  |  | <0.001 |  |  |  | 0.058 |
| I | 10(3.57%) | 14(5.00%) |  |  | 10(4.02%) | 13(5.22%) |  |
| II | 71(25.36%) | 87(31.07%) |  |  | 68(27.31%) | 76(30.52%) |  |
| III/IV | 194(69.29%) | 152(54.29%) |  |  | 168(67.47%) | 148(59.44%) |  |
| Unknown | 5(1.78%) | 27(9.64%) |  |  | 3(1.20%) | 12(4.82%) |  |
| N stage |  |  | <0.001 |  |  |  | 1.000 |
| N0 | 44(15.71%) | 62(22.14%) |  |  | 44(17.67%) | 44(17.67%) |  |
| N1 | 11(3.93%) | 16(5.71%) |  |  | 6(2.41%) | 6(2.41%) |  |
| N2 | 2(0.71%) | 5(1.79%) |  |  | 2(0.80%) | 2(0.80%) |  |
| N3 | 5(1.79%) | 2(0.71%) |  |  | 2(0.80%) | 2(0.80%) |  |
| Nx | 218(77.86%) | 195(69.65%) |  |  | 195(78.32%) | 195(78.32%) |  |
| RNE |  |  | <0.001 |  |  |  | 0.160 |
| <15 | 125(44.64%) | 176(62.86%) |  |  | 125(50.20%) | 146(58.63%) |  |
| ≥15 | 150(53.57%) | 98(35.00%) |  |  | 119(47.79%) | 98(39.36%) |  |
| Unknown | 5(1.79%) | 6(2.14%) |  |  | 5(2.01%) | 5(2.01%) |  |
| Tumor size |  |  | <0.001 |  |  |  | 0.059 |
| <3cm | 7(2.50%) | 11(3.93%) |  |  | 6(2.41%) | 10(4.02%) |  |
| ≥3cm and <5cm | 105(37.50%) | 107(38.21%) |  |  | 105(42.17%) | 107(42.97%) |  |
| ≥5cm | 120(42.86%) | 88(31.43%) |  |  | 91(36.55%) | 88(35.34%) |  |
| Unknown | 48(17.14%) | 74(26.43%) |  |  | 47(18.87%) | 44(17.67%) |  |

Abbreviations PSM: Propensity score matching; RNE: Regional nodes examined
